# Supplementary figures and images for: Structures of Foot-and-mouth Disease Virus with neutralizing antibodies derived from recovered natural host reveal a mechanism for cross-serotype neutralization
Source: PLoS Pathog. 2021 Apr 28;17(4):e1009507. doi: 10.1371/journal.ppat.1009507 (PMC8081260; doi:10.1371/journal.ppat.1009507)

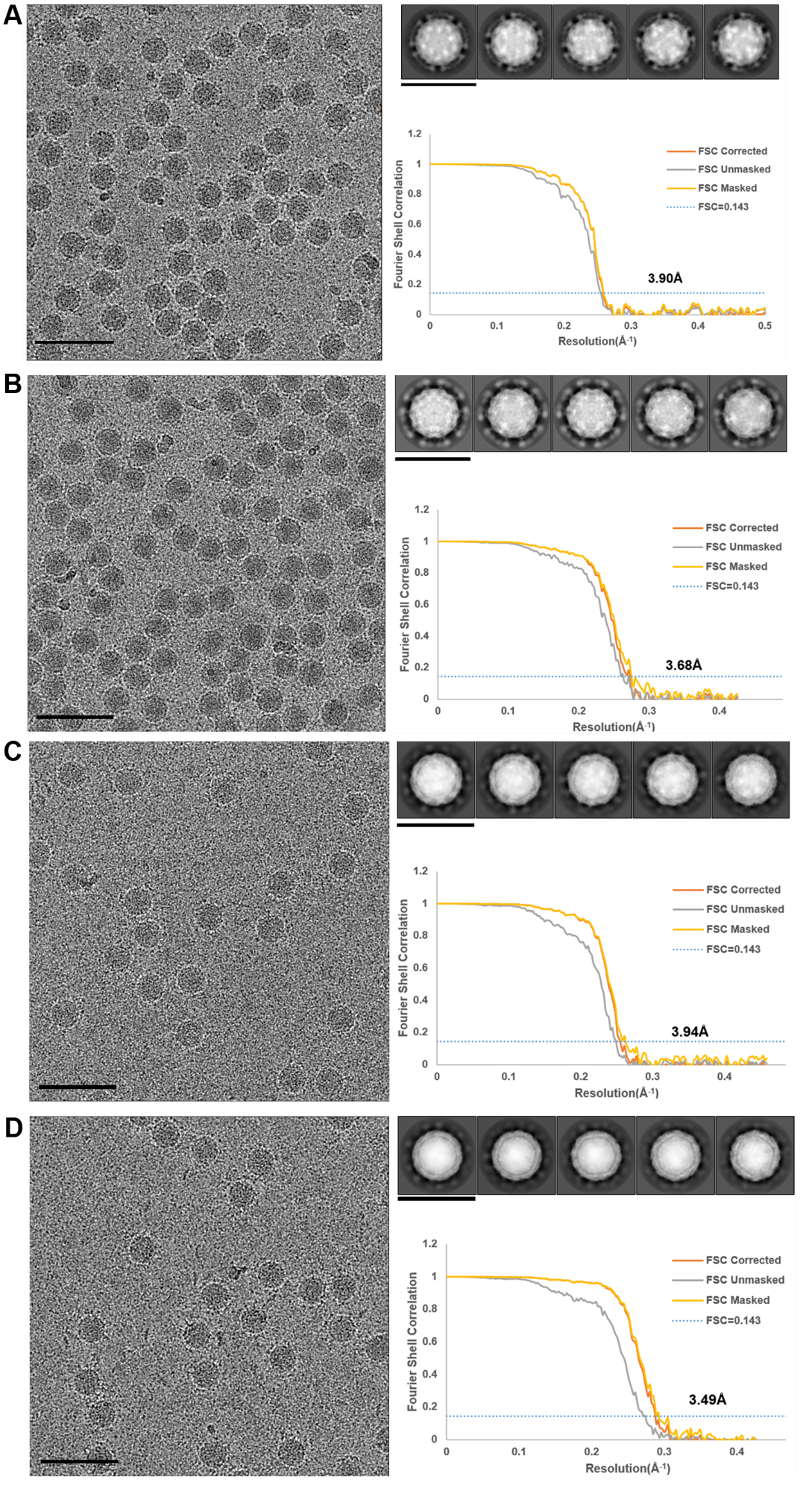

Supplement: S1 Fig — Typical electron micrographs were collected with a defocus of 2.0 μm (FMDV-OTi-B77), 1.9 μm (FMDV-OTi-F145), 1.5 μm (FMDV-OTi-R50) and 1.7 μm (FMDV-AWH-R50) (Scale bar, 1000 Å). Selected 2D class averages both show prominent spikes on the outer surface of viral particles (Scale bar, 480 Å). Fourier shell correlation (FSC) of the final 3D reconstruction after gold-standard refinement using RELION and THUNDER. The resolution corresponding to an FSC of 0.143 is shown for these virus-antibody complexes. FSC curves are plotted before (gray) and after (yellow) masking in addition to post correction (orange), accounting for the effect of the mask using phase randomization. (TIF) [file ppat.1009507.s001.tif]

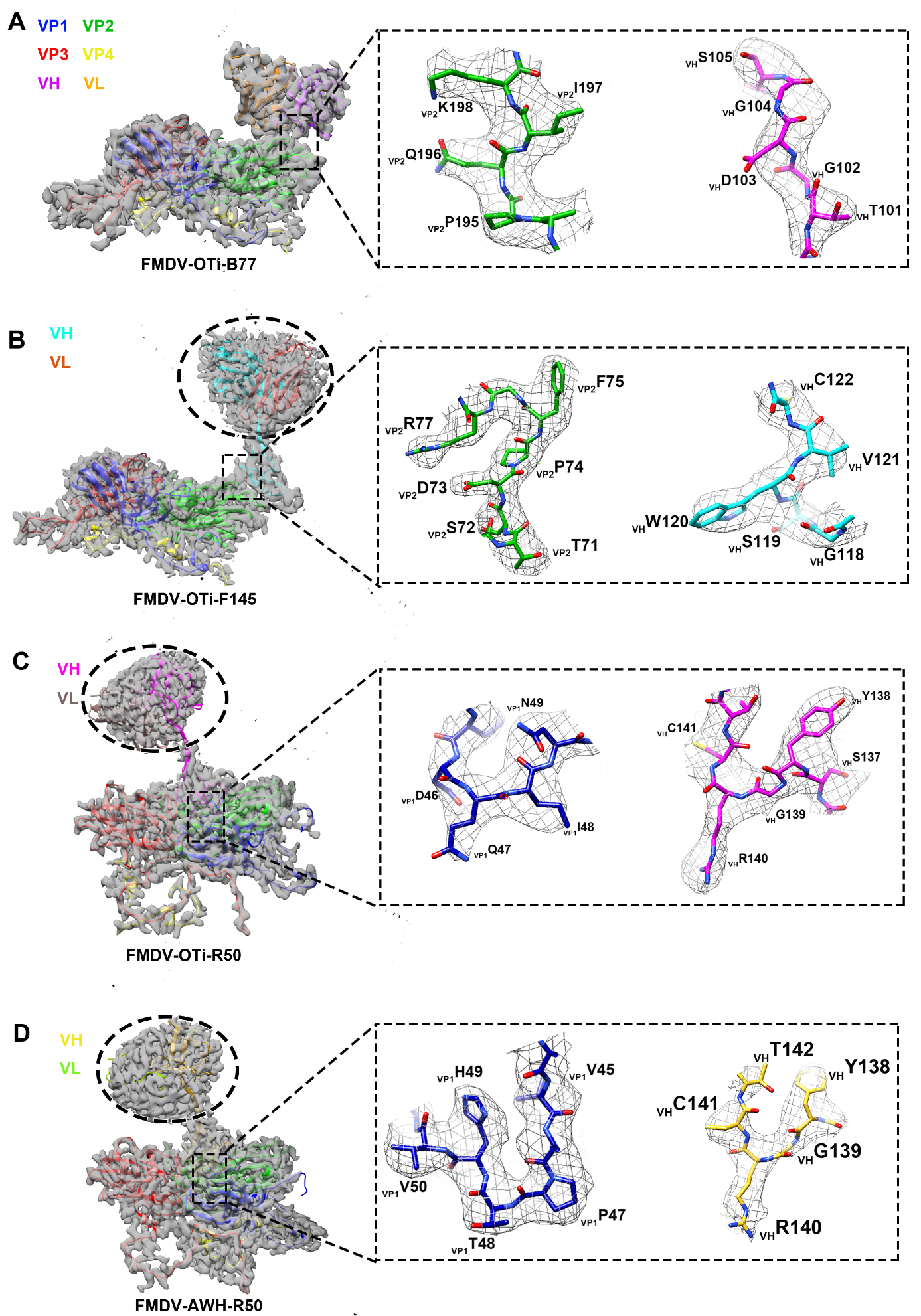

Supplement: S2 Fig — Surface representation of the density maps for a protomer of these complexes. VP1, VP2, VP3 and VP4 of the protomer are blue, green, red and yellow; VH and VL of B77 are purple and orange; and VH and VL of F145 are cyan and brown, respectively. The VH and VL of R50(FMDV-OTi-R50) are magenta and dark violet, and the VH and VL of R50(FMDV-AWH-R50) are yellow orange and limon, respectively. In the right panel, atomic models shown as sticks are superimposed to indicate the representative regions in wire frames. In the stick models, the residue numbers are indicated. The VP1, VP2 and VH residues are labeled with a subscript. Black dashed circles show the densities of the bound NAbs away from the contact surface. The densities are not sufficiently clear to trace all main chains. (TIF) [file ppat.1009507.s002.tif]

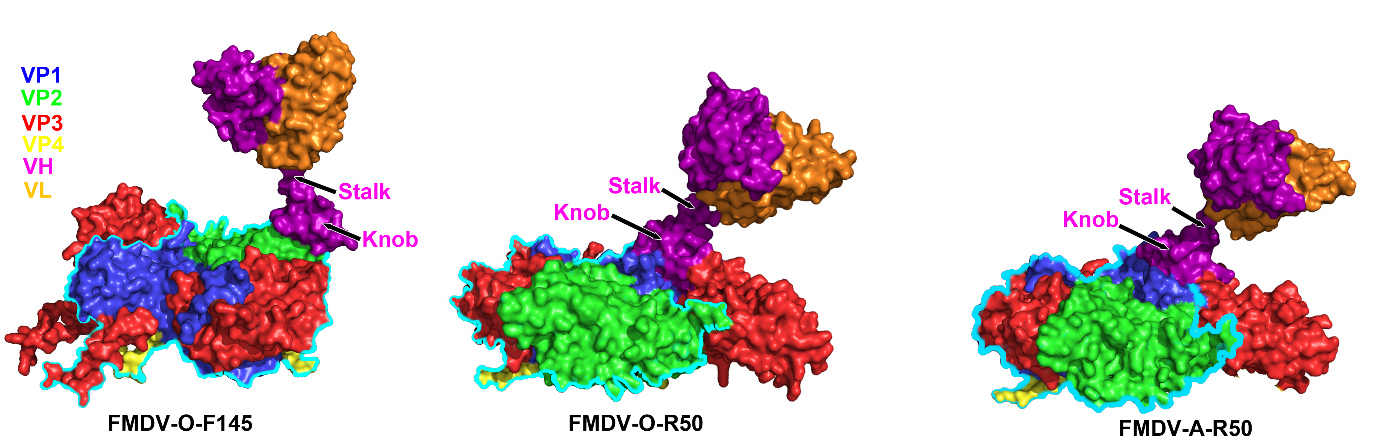

Supplement: S3 Fig — Structures of the FMDV-OTi-F145 complex, FMDV-OTi-R50 complex and FMDV-AWH-R50 complex are shown in surface representation. The border of one protomer is indicated by a sky-blue line. The VP1, VP2, VP3 and VP4 of the protomer are shown in blue, green, red and yellow, while the VH and VL are shown in purple and orange, respectively. (TIF) [file ppat.1009507.s003.tif]

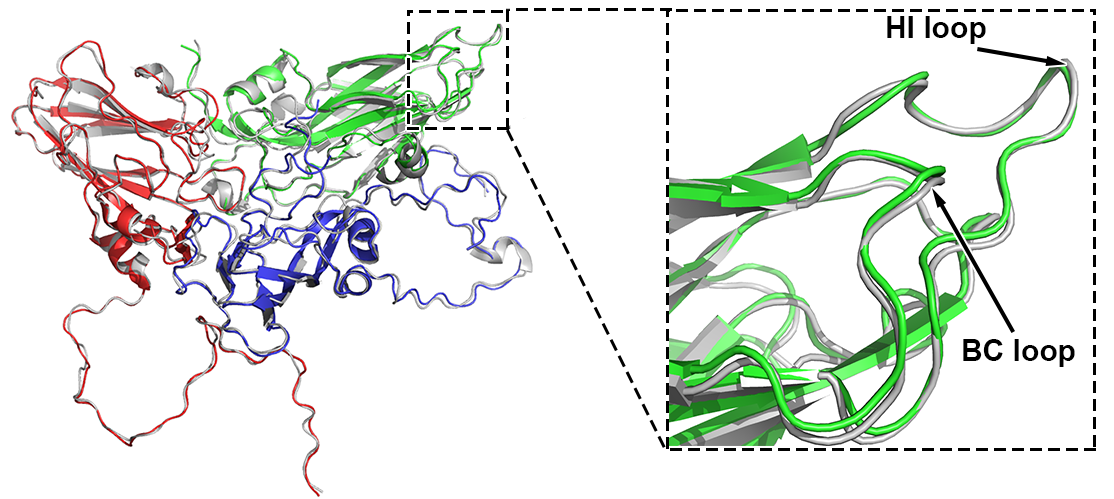

Supplement: S4 Fig — Protomers of FMDV-OTi (VP1: blue, VP2: green, VP3: red) and FMDV-AWH (gray) are aligned and shown as ribbon diagram in the same orientation. The VP2 BC-loop and HI-loop are framed and shown in a close-up view in the right panel. (TIF) [file ppat.1009507.s004.tif]

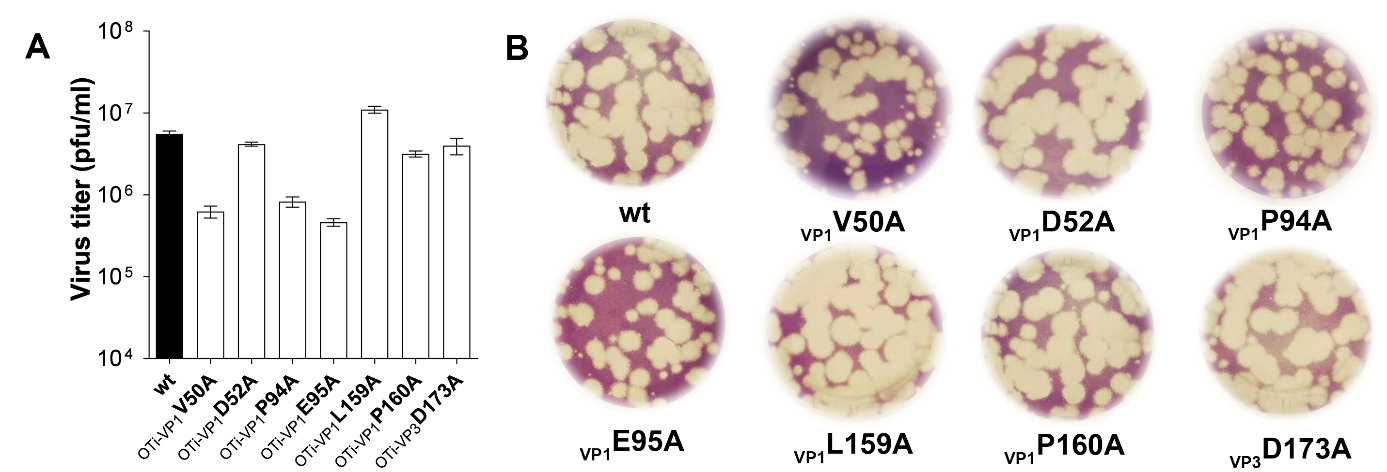

Supplement: S5 Fig — (A) Titrations of rescued viruses with indicated mutations in the virus-antibody interface. The data are shown as the mean of triplicates with S.D. (B) Plaques formed in BHK-21 cells by wild-type and mutants. The patterns of CPE correlated with the plaque size. (TIF) [file ppat.1009507.s005.tif]

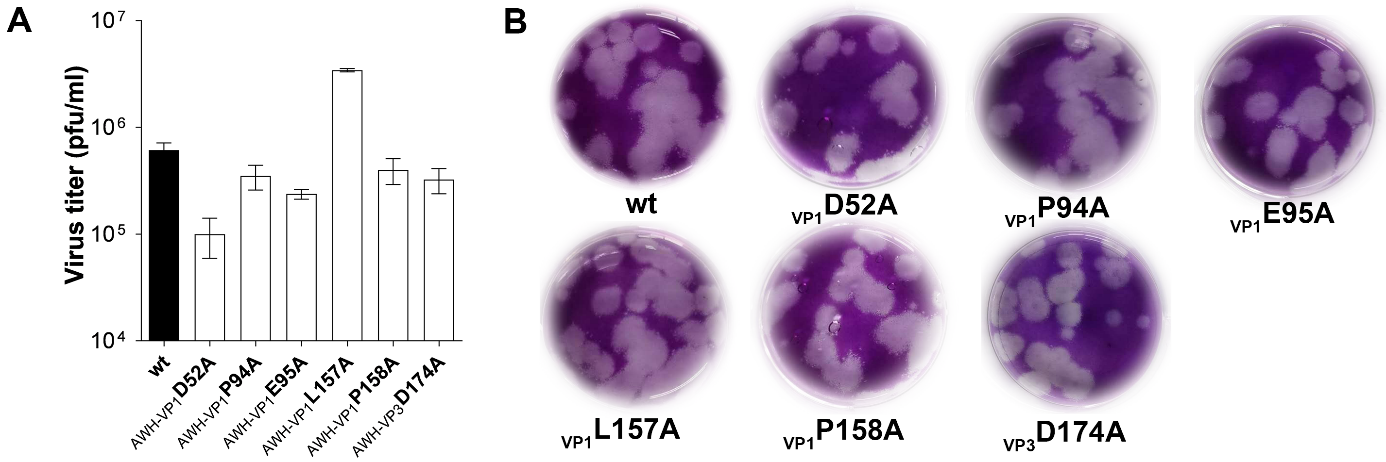

Supplement: S6 Fig — (A) Titrations of rescued viruses with indicated mutations in the virus-antibody interface. The data are shown as the mean of triplicates with S.D. (B) Plaques formed in BHK-21 cells by wild-type and mutants. The patterns of CPE correlated with the plaque size. (TIF) [file ppat.1009507.s006.tif]

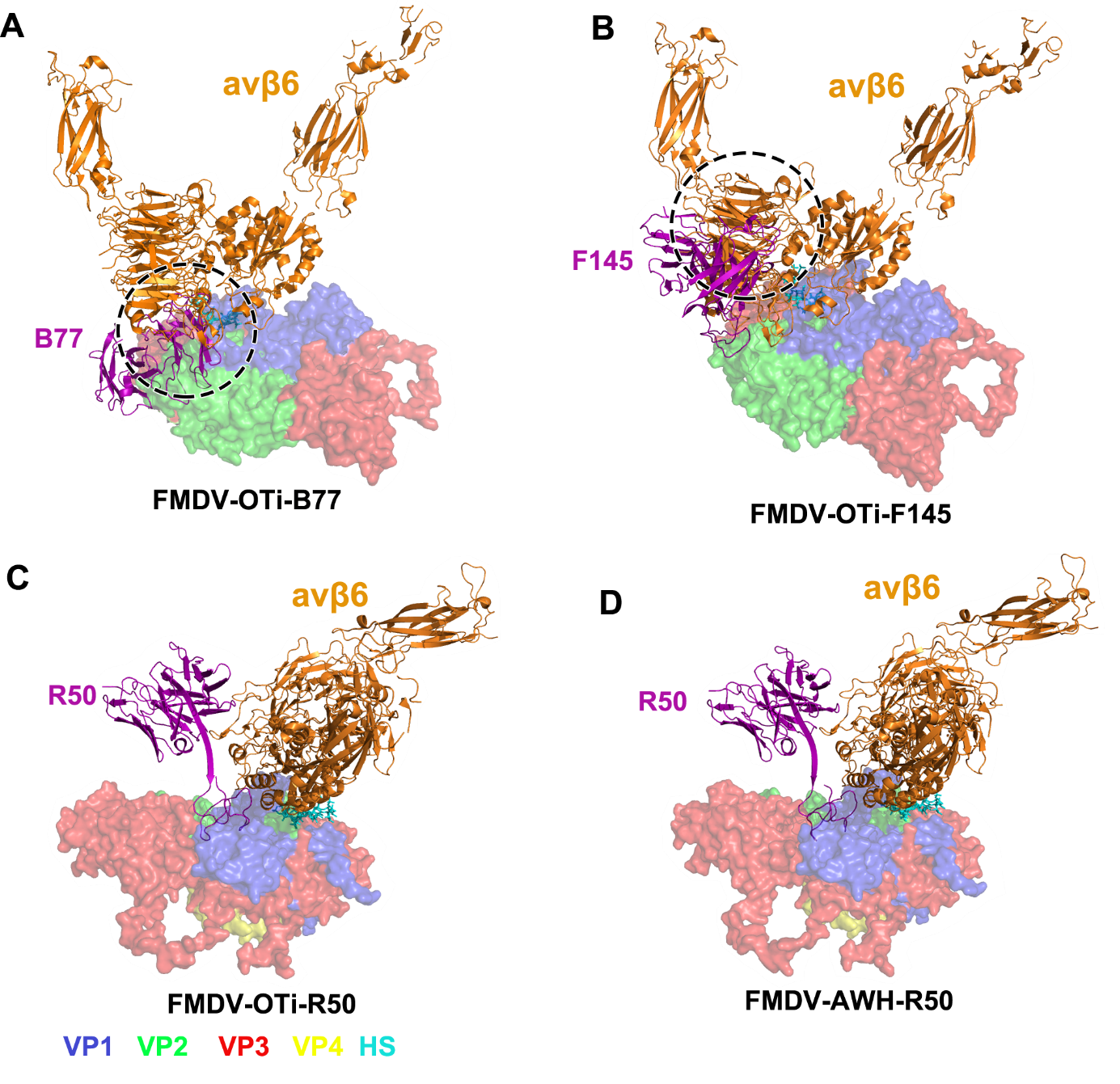

Supplement: S7 Fig — Binding modes of receptor [integrin (avβ6) and heparin sulfate (HS)] and scFv antibody B77 (A), F145 (B) and R50 (C, D). The panel shows a view down onto the capsid surface. VP1, VP2, VP3 and VP4 of the protomer are blue, green, red and yellow, respectively. The integrin (avβ6) and antibodies (B77, F145 and R50) are drawn in ribbon diagram; integrin (avβ6) is orange and antibodies (B77, F145 and R50) are purple. The heparin sulfate (HS) is drawn in cyan stick representation. Black dashed circles show significant clashes between antibody (B77 and F145) and integrin receptor. (TIF) [file ppat.1009507.s007.tif]

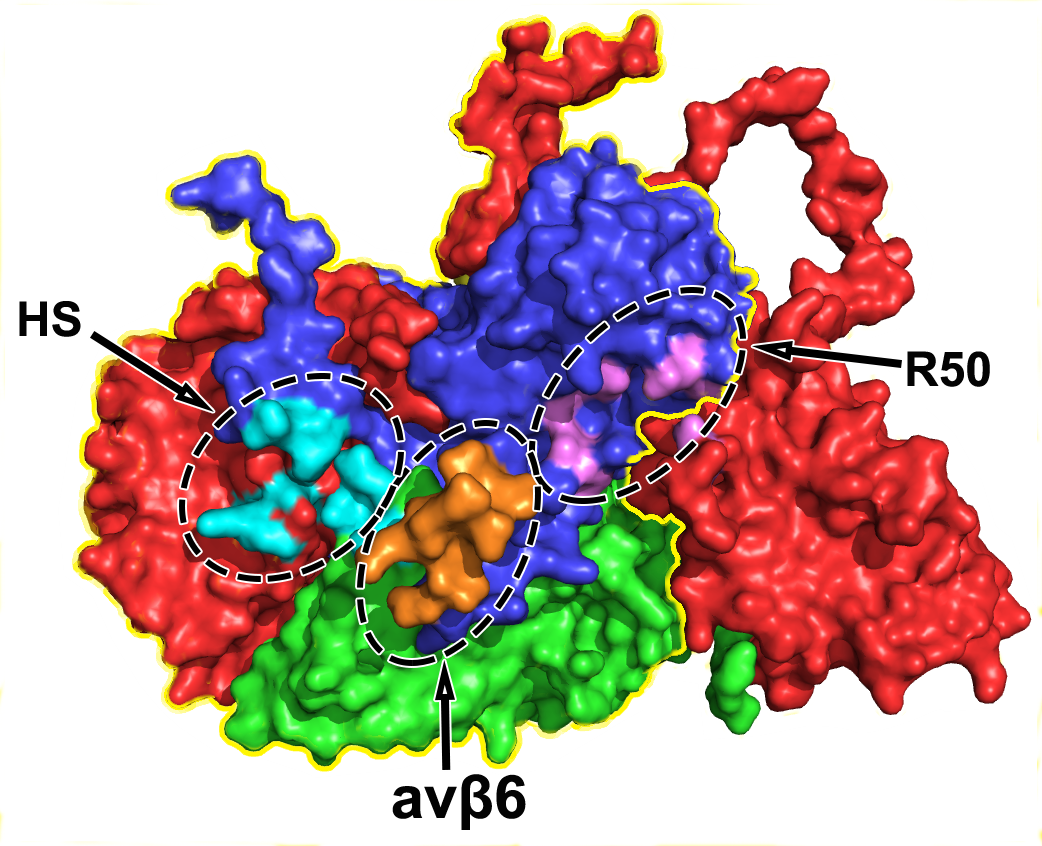

Supplement: S8 Fig — Residues identified for R50 are indicated in magenta, and residues identified for heparin sulfate (HS) and integrin (generally avβ6) are indicated in cyan and orange, respectively. The border of one protomer is indicated by a yellow line. (TIF) [file ppat.1009507.s008.tif]

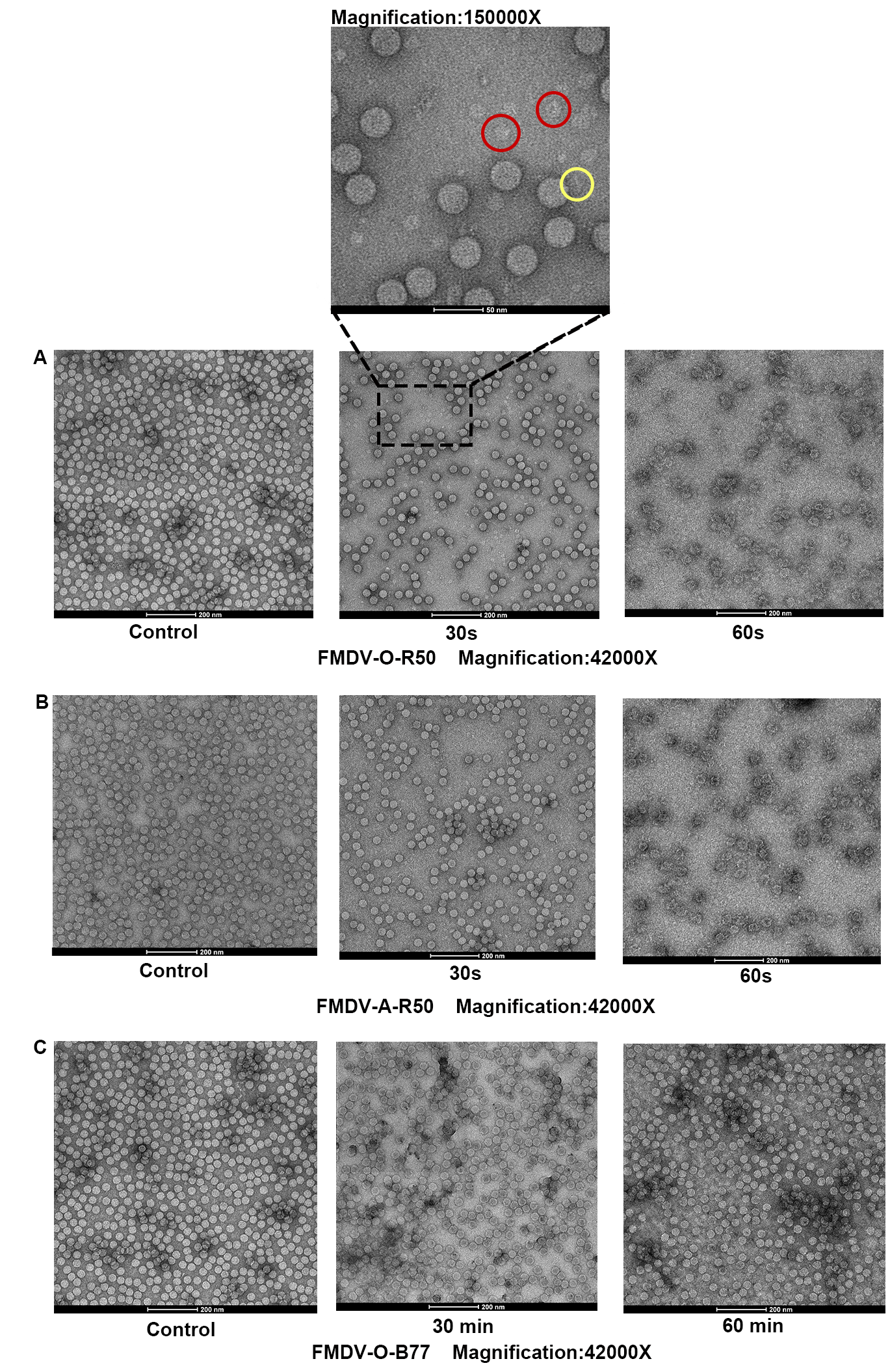

Supplement: S9 Fig — (A, B) Negative stain EM analysis of FMDV-OTi (A) or FMDV-AWH particles when incubated with R50 at 4°C for 30s or 60s. Red circle: pentamer, top view. Yellow circle: pentamer, side view. (C) Negative stain EM analysis of FMDV-OTi particles when incubated with B77 at 4°C for 30 min or 60 min. (TIF) [file ppat.1009507.s009.tif]

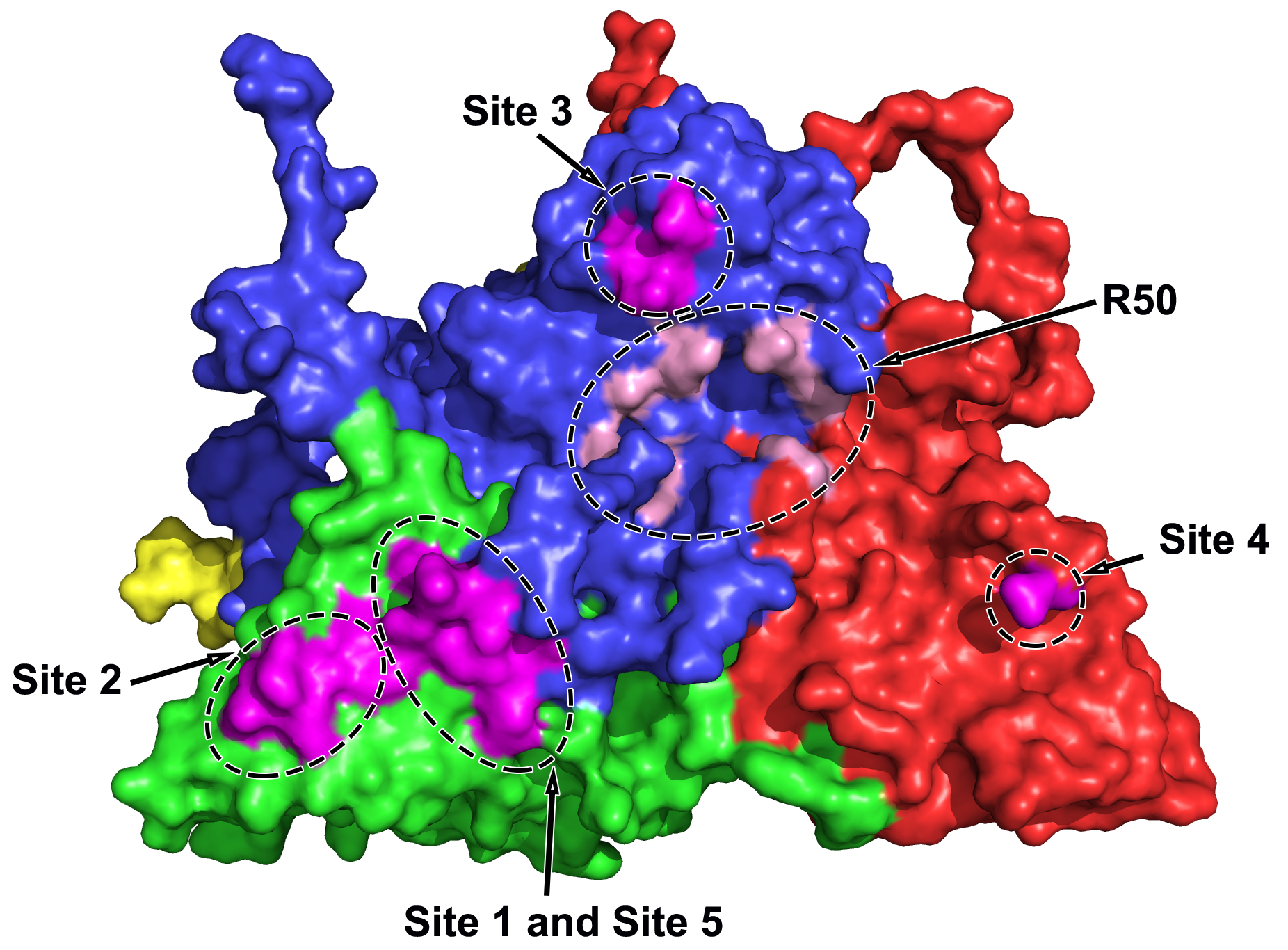

Supplement: S10 Fig — Sites 1–5 identified for murine monoclonal antibodies are indicated in magenta; residues identified for R50 are indicated in pink. (TIF) [file ppat.1009507.s010.tif]
