# Supplementary material for: Structures of Foot-and-mouth Disease Virus with neutralizing antibodies derived from recovered natural host reveal a mechanism for cross-serotype neutralization
Source: PLoS Pathog. 2021 Apr 28;17(4):e1009507. doi: 10.1371/journal.ppat.1009507 (PMC8081260; doi:10.1371/journal.ppat.1009507)
Supplement: S1 Table — (DOCX) [file ppat.1009507.s011.docx]

**S1 Table. Nested PCR primers used for amplifying variable regions of cattle IgG**

| **Primer** | **^a^Sequence** | **^b^Ta(**^◦^C) |
| --- | --- | --- |
| Ig λ chain outer-Forward: | CACCATGGCCTGGTCCCCTCTG | 56 |
| Ig λ chain outer-Reverse: | AAGTCGCTGATGAGACACACC | 56 |
| Ig λ chain inner-Forward: | TGGGCCCAGGCTGTRCTG | 55 |
| Ig λ chain inner-Reverse: | GCGGGAACAGGGTGACCGAG | 55 |
| Ig γ chain outer-Forward: | CCCTCCTCTTTGTGCTSTCAGCCC | 58/60 |
| Ig γ chain outer-Reverse: | GTCACCATGCTGCTGAGAGA | 60 |
| Ig γ chain inner-Forward: | AGAGGRGTYBTGTCCCAGG | 55 |
| Ig γ chain inner-Reverse: | CTTTCGGGGCTGTGGTGGAGGC | 55 |

^a^Degenerate bases were synthesized in these sequences, including S= C or G, Y=C or T, and R=A or G. ^b^Annealing temperature.
