# Supplementary material for: Structures of Foot-and-mouth Disease Virus with neutralizing antibodies derived from recovered natural host reveal a mechanism for cross-serotype neutralization
Source: PLoS Pathog. 2021 Apr 28;17(4):e1009507. doi: 10.1371/journal.ppat.1009507 (PMC8081260; doi:10.1371/journal.ppat.1009507)
Supplement: S2 Table — (DOCX) [file ppat.1009507.s012.docx]

**S2 Table. Cryo-EM data collection and refinement statistics**

|  | **FMDV-OTi-B77** | **FMDV-OTi-F145** | **FMDV-OTi-R50** | **FMDV-AWH-R50** |
| --- | --- | --- | --- | --- |
| **Data collection and processing** |  |  |  |  |
| Magnification | 110,000 | 110,000 | 110,000 | 110,000 |
| Voltage (kV) | 200 | 200 | 200 | 200 |
| Electron exposure (e–/Å^2^) | 25 | 25 | 25 | 25 |
| Defocus range (μm) | -2.4 to -1.4 | -2.4 to -1.4 | -2.4 to -1.4 | -2.4 to -1.4 |
| Pixel size (Å) | 0.932 | 0.932 | 0.932 | 0.932 |
| Symmetry imposed | I1 | I1 | I1 | I1 |
| Initial particle images (no.) | 25580 | 25385 | 19421 | 30169 |
| Final particle images (no.) | 15070 | 11054 | 15499 | 12644 |
| Map resolution (Å)  FSC threshold | 3.90  0.143 | 3.68  0.143 | 3.94  0.143 | 3.49  0.143 |
| Map resolution range (Å) | 3.2-4.8 | 3.0-10.0 | 3.2-10.0 | 3.0-10.0 |
|  |  |  |  |  |
| **Refinement** |  |  |  |  |
| Initial model used (PDB code) | 1BBT;  5YAX | 1BBT;  6e9u | 1BBT;  6e9u | 1BBT;  6e9u |
| Model resolution (Å)  FSC threshold | 2.60;2.50  0.143 | 2.60;2.29  0.143 | 2.60;2.29  0.143 | 2.60;2.29  0.143 |
| Model resolution range (Å) | ꝏ to 2.60 | ꝏ to 2.60 | ꝏ to 2.60 | ꝏ to 2.60 |
| Map sharpening *B* factor (Å^2^) | -267 | -237 | -267 | -197 |
| Model composition  Non-hydrogen atoms  Protein residues  Ligands | 6572  853  0 | 6848  898  0 | 6944  909  0 | 6975  909  0 |
| *B* factors (Å^2^)  Protein  Ligand | 34.18  -- | 13.24  -- | 25.14  -- | 24.86  -- |
| R.m.s. deviations  Bond lengths (Å)  Bond angles (°) | 0.011  0.944 | 0.007  0.898 | 0.010  1.010 | 0.008  0.974 |
| Validation  MolProbity score  Clashscore  Poor rotamers (%) | 2.87  6.93  0.06 | 2.33  5.14  0.00 | 2.52  6.04  0.00 | 3.22  5.95  0.00 |
| Ramachandran plot  Favored (%)  Allowed (%)  Disallowed (%) | 89.42  10.28  0.30 | 92.40  7.60  0.00 | 90.37  9.63  0.00 | 90.70  9.30  0.00 |
