# Supplementary material for: Structures of Foot-and-mouth Disease Virus with neutralizing antibodies derived from recovered natural host reveal a mechanism for cross-serotype neutralization
Source: PLoS Pathog. 2021 Apr 28;17(4):e1009507. doi: 10.1371/journal.ppat.1009507 (PMC8081260; doi:10.1371/journal.ppat.1009507)
Supplement: S3 Table — (DOCX) [file ppat.1009507.s013.docx]

**S3 Table. HCDR3 amino acid sequences of bovine antibodies**

| **ScFv** | **HCDR3 Length (aa)** | **HCDR3 amino acid sequence** | |
| --- | --- | --- | --- |
| B77 | 18 | | A^96^KSRYTGDGSIGLYGVDA^113^ |
| F145 | 50 | | T^96^TVYHETSRTCPDGYIYDPGCGGSWVCSRLFPTDRCIVGRTTTYE  WYVDA^145^ |
| R50 | 61 | | A^117^TVHQHTSEKRTCPRAYRPDCAARWDCPGGADCGYCNFGAGSY  GRCTPFTLTY TFENYVHT^177^ |
