# Supplementary material for: Structures of Foot-and-mouth Disease Virus with neutralizing antibodies derived from recovered natural host reveal a mechanism for cross-serotype neutralization
Source: PLoS Pathog. 2021 Apr 28;17(4):e1009507. doi: 10.1371/journal.ppat.1009507 (PMC8081260; doi:10.1371/journal.ppat.1009507)
Supplement: S4 Table — (DOCX) [file ppat.1009507.s014.docx]

**S4 Table. FMDV-OTi-B77 interaction residues**

| Domain | Residue | Distance (Å) | B77 | CDR |
| --- | --- | --- | --- | --- |
| VP2 βB | H65(NE2) | 3.15 | S95(OG) | LCDR3 |
| VP2 BC-Loop | D68(OD2) | 2.72 | W93(CZ3) | LCDR3 |
|  | D68(CG) | 3.51 | W93(CZ2) | LCDR3 |
|  | V70(CG1) | 3.74 | L108(CD2) | HCDR3 |
|  | V70(CG2) | 3.27 | L108(CD2) | HCDR3 |
|  | T71(OG1) | 2.8 | T101(OG1) | HCDR3 |
|  | S72(OG) | 2.69 | W114(NE1) | HCDR3 |
|  | R77(NH2) | 3.28 | G32(O) | LCDR1 |
|  | Y79(O) | 3.11 | T31(OG1) | LCDR1 |
|  | N190(ND2) | 3.3 | M56(SD) | HCDR2 |
| VP2 HI-Loop | Q196(OE1) | 2.32 | S105(OG) | HCDR3 |
|  | Q196(NE2) | 2.85 | S105(OG) | HCDR3 |

The interaction residues were computed using the CCP4 hydrogen bond distance cutoff of 4.0 Å and the salt-bridge distance cutoff of 4.0 Å. The red font refers to a hydrogen bond or salt-bridge between the amino-acid side chain and side chain.

.
