# Supplementary material for: Structures of Foot-and-mouth Disease Virus with neutralizing antibodies derived from recovered natural host reveal a mechanism for cross-serotype neutralization
Source: PLoS Pathog. 2021 Apr 28;17(4):e1009507. doi: 10.1371/journal.ppat.1009507 (PMC8081260; doi:10.1371/journal.ppat.1009507)
Supplement: S5 Table — (DOCX) [file ppat.1009507.s015.docx]

**S5 Table. FMDV-OTi-F145 interaction residues**

| Domain | Residue | Distance (Å) | F145 | CDR |
| --- | --- | --- | --- | --- |
| VP2 BC-Loop | V70(CG2) | 3.20 | G118(O) | HCDR3 |
|  | T71(CG2) | 3.84 | W120(CB) | HCDR3 |
|  | T71(OG1) | 3.79 | C131(O) | HCDR3 |
|  | T71(CG2) | 3.78 | C131(CB) | HCDR3 |
|  | S72(OG) | 3.33 | S119(OG) | HCDR3 |
| VP2 HI-Loop | V189(CG1) | 3.68 | R130(CB) | HCDR3 |
|  | V189(CG2) | 3.34 | R130(O) | HCDR3 |
|  | N190(OD1) | 3.81 | D129(OD1) | HCDR3 |
|  | T191(OG1) | 3.98 | T128(OG1) | HCDR3 |
|  | P195(O) | 3.36 | C116(CB) | HCDR3 |
|  | Q196(OE1) | 3.80 | G117(N) | HCDR3 |

The interaction residues were computed using the CCP4 hydrogen bond distance cutoff of 4.0 Å and the salt-bridge distance cutoff of 4.0 Å. The red font refers to a hydrogen bond or salt-bridge between the amino-acid side chain and side chain.
