# Supplementary material for: Structures of Foot-and-mouth Disease Virus with neutralizing antibodies derived from recovered natural host reveal a mechanism for cross-serotype neutralization
Source: PLoS Pathog. 2021 Apr 28;17(4):e1009507. doi: 10.1371/journal.ppat.1009507 (PMC8081260; doi:10.1371/journal.ppat.1009507)
Supplement: S6 Table — (DOCX) [file ppat.1009507.s016.docx]

**S6 Table. FMDV-OTi-R50 interaction residues**

| Domain | Residue | Distance (Å) | R50 | CDR |
| --- | --- | --- | --- | --- |
| VP1 BC-Loop | V50(CG2) | 3.87 | W120(NE1) | HCDR3 |
|  | D52(OD2) | 2.29 | R140(NH2) | HCDR3 |
|  | D52(OD2) | 3.38 | R140(NH1) | HCDR3 |
|  | Q55(NE2) | 3.22 | R119(O) | HCDR3 |
| VP1 EF-Loop | G92(O) | 3.84 | F133(O) | HCDR3 |
|  | A93(O) | 3.03 | F133(O) | HCDR3 |
|  | P94(O) | 3.98 | N132(CG) | HCDR3 |
|  | E95(OE1) | 3.20 | R119(NH2) | HCDR3 |
| VP1 GH-Loop | R157(NH1) | 3.95 | T142(OG1) | HCDR3 |
|  | A158(O) | 3.98 | R140(N) | HCDR3 |
|  | L159(CD1) | 2.70 | R140(NH1) | HCDR3 |
|  | P160(O) | 3.74 | R140(N) | HCDR3 |
|  | T161(CG2) | 3.95 | R140(CB) | HCDR3 |
| VP3 GH-Loop | D173(OD2) | 3.00 | Y130(OH) | HCDR3 |
|  | A174(O) | 3.63 | R110(NH1) | HCDR3 |
|  | A174(CB) | 3.26 | R110(NH1) | HCDR3 |
|  | A175(CA) | 3.66 | R110(NH1) | HCDR3 |
|  | A175(O) | 3.35 | R110(NH1) | HCDR3 |
|  | E176(CG) | 3.36 | S137(OG) | HCDR3 |
|  | T177(OG1) | 2.80 | Y138(OH) | HCDR3 |

The interaction residues were computed using the CCP4 hydrogen bond distance cutoff of 4.0 Å and the salt-bridge distance cutoff of 4.0 Å. The red font refers to a hydrogen bond or salt-bridge between the amino-acid side chain and side chain.
