# Supplementary material for: Structures of Foot-and-mouth Disease Virus with neutralizing antibodies derived from recovered natural host reveal a mechanism for cross-serotype neutralization
Source: PLoS Pathog. 2021 Apr 28;17(4):e1009507. doi: 10.1371/journal.ppat.1009507 (PMC8081260; doi:10.1371/journal.ppat.1009507)
Supplement: S7 Table — (DOCX) [file ppat.1009507.s017.docx]

**S7 Table. FMDV-AWH-R50 interaction residues**

| Domain | Residue | Distance (Å) | R50 | CDR |
| --- | --- | --- | --- | --- |
| VP1 BC-Loop | V50(CG1) | 3.84 | W120(NE1) | HCDR3 |
|  | D52(OD2) | 3.56 | R140(NH2) | HCDR3 |
|  | D52(OD2) | 3.89 | R140(NH1) | HCDR3 |
|  | Q55(NE2) | 3.23 | R119(NH2) | HCDR3 |
| VP1 EF-Loop | G92(O) | 3.76 | F133(O) | HCDR3 |
|  | A93(N) | 3.16 | F133(O) | HCDR3 |
|  | P94(O) | 3.92 | N132(CG) | HCDR3 |
|  | E95(OE1) | 2.79 | R119(NH2) | HCDR3 |
| VP1 GH-Loop | Q156(OE1) | 3.55 | D121(OD1) | HCDR3 |
|  | Q156(NE1) | 2.2 | D121(OD1) | HCDR3 |
|  | L157(O) | 3.71 | R140(NE) | HCDR3 |
|  | P158(O) | 3.04 | R140(NH1) | HCDR3 |
|  | A159(O) | 3.60 | W120(CZ2) | HCDR3 |
| VP3 GH-Loop | D174(OD2) | 3.04 | Y130(OH) | HCDR3 |
|  | V175(CG1) | 3.39 | S137(OG) | HCDR3 |
|  | A176(CB) | 3.67 | S137(OG) | HCDR3 |
|  | D177(N) | 3.74 | S137(OG) | HCDR3 |

The interaction residues were computed using the CCP4 hydrogen bond distance cutoff of 4.0 Å and the salt-bridge distance cutoff of 4.0 Å. The red font refers to a hydrogen bond or salt-bridge between the amino-acid side chain and side chain.
