# Supplementary material for: Structures of Foot-and-mouth Disease Virus with neutralizing antibodies derived from recovered natural host reveal a mechanism for cross-serotype neutralization
Source: PLoS Pathog. 2021 Apr 28;17(4):e1009507. doi: 10.1371/journal.ppat.1009507 (PMC8081260; doi:10.1371/journal.ppat.1009507)
Supplement: S8 Table — (DOCX) [file ppat.1009507.s018.docx]

**S8 Table. Selection of neutralization-resistant FMDV**

| Antibody | Parent virus | Mutation | Frequency | Neutralization  Concentration^a^  (µg/mg) |
| --- | --- | --- | --- | --- |
| B77 | O/Tibet/99 | _VP2_N190S | 5/5 | >400 |
| F145 | O/Tibet/99 | _VP2_ S72A | 3/3 | >400 |

^a^Neutralization concentration was determined as the lowest antibody concentration that protected cells from CPE.
