# Supplementary material for: Structures of Foot-and-mouth Disease Virus with neutralizing antibodies derived from recovered natural host reveal a mechanism for cross-serotype neutralization
Source: PLoS Pathog. 2021 Apr 28;17(4):e1009507. doi: 10.1371/journal.ppat.1009507 (PMC8081260; doi:10.1371/journal.ppat.1009507)
Supplement: Attachment — Submitted filename: review.docx [file ppat.1009507.s019.docx]

He et al. characterized foot-and-mouth disease virus (FMDV) and three neutralizing antibodies (NAbs). The authors isolated two NAbs for FMDV strain O and isolated one NAb that is cross-reactive for strains O and A. By means of cryogenic electron microscopy (cryo-EM), He et al. determined high-resolution structures of four FMDV-NAb complexes. They also performed neutralization experiments and generated neutralization escape mutants. Presumably to make experiments simpler, they made and used single-chain fragment variable antibodies. The two O-strain NAbs (B77 and F145) bound FMDV near the 3-fold symmetry axis of the capsid. The O/A cross-binding NAb (R50) bound FMDV near the 5-fold symmetry axis. R50 bound most of the same residues in both strains. Precise interactions of the NAbs and FMDV were mapped. Escape mutants were successfully produced. The authors proposed neutralizing mechanisms for all three NAbs. The analysis is thorough and rigorous. Structural experiments were complemented by mutational experiments. Antibody-producing cells were obtained from an animal by an elaborate protocol. Conclusions are backed up by substantial evidence. I found the work enlightening and a step forward for the field. This work has implications not only for FMDV work, but also for virus-antibody studies in general. It shows an example of how a cross-serotype neutralizing antibody binds to a virus. At least two different neutralizing mechanisms appear to be at work because the R50 caused different effects on FMDV than did the other two NAbs. I recommend publication but suggest that the following corrections be made.

He al. proposed mechanisms for how the three NAbs neutralize FMDV. B77 and F145 are proposed to block receptor binding, and R50 is proposed to cause the FMDV capsid to fall apart. The latter was discovered through an interesting observation by the authors. Treatment of FMDV with R50 resulted in dissociation of the particles. I have several questions:

-For picornavirus capsids, a "promoter" was defined as one closely associated VP1-4 subunits and is considered an assembly unit (5 protomers form a pentamer, then 12 pentamers form a capsid). I note that the R50 interaction is an inter-protomer interaction (Figs. S8 and S10). The authors should mention this. Does that fact have any implications for the mechanism of neutralization?

-Yes, the binding of B77 or F145 will occlude the receptor binding site. But in vivo, one is not likely to have all 60 sites occupied. Therefore, presumably the receptor could simply bind to an unoccupied site. Also, in vivo the antibody will be an IgG or some other multi-site entity. What alternative mechanism does these facts suggest? Could antibody binding prevent conformational changes during disassembly/uncoating as was hypothesized for a neutralizing antibody to poliovirus?

-Are the B77 and F145 interactions inter- or intra-protomer?

-The dissociation might explain the lower particle density seen in the micrographs of frozen FMDV-R50 complexes (Fig. S1). Cryo-samples of FMDV-R50 were prepared after 30 sec incubation. Is the lower density in the micrographs further evidence of the dissociation?

-In those cryogenic-samples were pentamer-R50 or pentamer particles seen? Does R50 retain affinity after the dissociation?

The authors note five known FMDV neutralizing antigen sites on FMDV (pp. 12-13). The R50 binding site appears to be a new site. This should be stated. Do B77 and F145 bind to one of the five sites or to a different site? The five sites are all intra-protomer sites. The R50 site is novel in that it overlaps the protomer boundary.

The A serotypes have a proline at residue 47 of VP1 (Fig. 5E). The O serotypes have S or Q at that position. Do the prolines induce a different loop conformation for the A serotypes or is the conformation similar despite the presence of a proline in the A serotypes? Apparently the conserved residues at 50 and 52 still bind to the antibody.

A figure in the main part of the paper that shows epitopes on unbound FMDV would be very helpful. I recommend showing FMDV capsid proteins as ribbons.

I suggest the NAb footprints, now shown in Fig. S3, be put into Fig. 2.

Many details of the FMDV-NAb interactions are written out in the text (pp. 7-11). Much of this would be clearer if it were put in a table and the pertinent messages about the interactions described in the text.

In Tables S4-S7, interacting residues are given for all four complexes. In each case, red designates "a hydrogen bond or salt-bridge". What are the black interactions? Many or most of the black ones are polar residues (N or O) and the distance is less than 4 angs. Seemingly these would also qualify for being H-bonds or "salt" interactions?

I was confused by the description of the grids that were used.

-"carbon-coated copper grid" was stated but in parentheses was stated "GIG Au 1.2/1/3". Was gold or copper used?

-I suggest instead of "Lantuo" that the full company name be stated for the benefit of those who are unfamiliar with that company. (This was the first time I had heard of it.)

He et al. state they used a calibrated magnification of 110kX. How was magnification calibrated? Was magnification refined with the crystal structure of FMDV that was fitted into the cryo-EM density maps?

Some figures are too small to see data clearly (e.g., Figs 3, S1, S2, S9).

The following terms or portions of the paper are unclear or need to be fixed:

-"quasi 5-fold axis" (p. 6), I know of no quasi 5-fold axis in picornaviruses.

-"the densities of the bound NAbs away from the contact surface were not sufficiently clear to trace all main chains (S2 Fig)." I do not see this illustrated or discussed in Fig S2.

-"The globular knob of F145 interacts with VP2, but the globular knob of R50 binds to a shallow depression on the capsid, contacting the two major capsid proteins VP1 and VP3 (Figs 2I-2L)." I do not see this in those panels. Perhaps a label or different figure is needed?

-Why is Fig. S4 referenced at the top of p. 8? It follows the comment ". . . is strictly conserved among serotype O".

-p. 8, line 198, "F45"

-p. 10, lines 252-254. The sentence here refers to Figs 5A-5D, but I don't see hydrogen bonds shown in those figures.

-p. 16, line 410, I think Supplemental Table 1 is what the authors intended.

-In Fig. S1, FSC Corrected, FSC Unmasked, and FSC Masked need to be explained.

-In Fig. S1, "Typical electron micrographs were collected with a defocus of ...." Specific defocus values for specific cryo-samples are given. This suggests all micrographs for each specified sample were collected only at those values. The methods says a range of defocus values were used.

-Fig. S3 "Roadmap" should be placed in parentheses because it is a jargon term. Was the Roadmap program used? No reference or explanation was given for how these diagrams were determined.

-In many places, authors call ribbon depictions of proteins "cartoons" or "cartoon representations". "Ribbon diagram" is a more accurate term.

-In caption for Fig. 1, "(B) . . . are green." Nothing in Fig. 1B is green.

-Figs. 4 and 5, "labels in the magenta regions" and "labels in the pink regions" is unclear wording.

-Fig. 5, "... indicated by blue and white ..." Do the authors mean light blue?
