# Supplementary material for: Structures of Foot-and-mouth Disease Virus with neutralizing antibodies derived from recovered natural host reveal a mechanism for cross-serotype neutralization
Source: PLoS Pathog. 2021 Apr 28;17(4):e1009507. doi: 10.1371/journal.ppat.1009507 (PMC8081260; doi:10.1371/journal.ppat.1009507)
Supplement: Attachment — Submitted filename: Response to reviewers.docx [file ppat.1009507.s020.docx]

Dear editors and reviewers:

Thank you for your letter and for the reviewers’ comments concerning our manuscript entitled “Structures of Foot-and-mouth Disease Virus with neutralizing antibodies derived from recovered natural host reveal a mechanism for cross-serotype neutralization”. Those comments are all valuable and very helpful for revising and improving our paper, as well as the important guiding significance to our researches. We have studied comments carefully and have made correction which we hope meet with approval. Revised portion are marked in yellow highlight in the paper. The main correction in the paper and the responds to reviewers’ comments are as following:

**Response to Reviewer #1:**

The authors do not show any neutralization assay for the new cross-reactive antibody to any virus. Levels of cross neutralization between multiple isolates in the O and A serotypes should be included. A binding antibody does not mean neutralization. The level of neutralization is also important. Does this cross-reactive antibody neutralize O serotypes at the same levels as the two previously identified antibody's? Does it neutralize the A serotype at the same level?
**Response:** To investigate the neutralization breadth and potency of R50 scFv antibody, we propagated, purified three representative strains of FMDV serotype O (O/Mya/98; O/HN/CHA/93; O/Tibet/99) obtained from three currently epidemic topotypes (SEA, Cathay, and ME-SA, respectively) and two representative strains of FMDV serotype A (A/WH/CHA/09; A/GDMM/CHA/2013) from the Asia topotypes SEA-97/G1 and G2 in China , and separately examined their binding and neutralization abilities by flow cytometry and microneutralization assay. The results were shown in Fig 1B and Fig 1C. Cross-reactive NAbs, in general, exhibit relatively weak antiviral activities than virus specific. Our results also show the B77 and F145(serotype-O specific neutralizing antibody) showed relatively high antiviral activities than R50(serotype O and A cross-reactive neutralizing antibody).

In the search for a universal FMDV vaccine as the authors describe, it is important that the side of binding is highly conserved among within serotype and between serotypes. Is the identified area conserved, and how high affinity does this antibody bind?

**Response:** The novel neutralizing antigenic site formed by _OTi-VP1_V50, _OTi-VP1_D52, _OTi-VP1_P94, _OTi-VP1_E95, _OTi-VP1_L159(or _AWH-VP1_L157), _OTi-VP1_P160 (or _AWH-VP1_P158) and _OTi-VP3_D173 (or _AWH-VP3_D174) is highly conserved among FMDV serotypes O and A and plays a major role in the induction of a neutralizing immune response (Fig 5E).

We evaluated the binding activity of R50 with FMDV serotype O and A strains. The results were shown in Fig 1B.

It would be good to see an isolate that doesn't have this region conserved as a control.

**Response:** we appreciate your comment and agree the conserved region isolate control would leverage the current result, but unfortunately, we have not obtain an isolate as a control.

The authors examine mutants in the ab-interface, most do not show a real decrease, with only a couple showing one log decrease. This is interesting, how do these mutants change binding affinity or neutralization using the new antibody?

**Response:** Although most of single mutants do not completely escape the antibody-mediated neutralization. But these mutants lowered the neutralizing ability compared with wild-type virus. Meanwhile, our study shows that R50 recognize conformational epitopes by western blot. These residues are both important in maintaining epitope conformation or function.

It would be interesting if this new antibody could be tested in an animal experiment to show antibody treatment can prevent infection for both A and O serotypes.

**Response:** Foot and mouth disease (FMD) is the first of the Class A infectious diseases in our country. Animal experiments on foot-and-mouth disease virus must be conducted in an animal biosafety level 3 laboratory. Due to the outbreak of the Coronavirus Disease of 2019 (COVID-19), other microbe and viruses experiments required BSL3 laboratory have been postponed. We have submitted an application, but have not gotten approved till now.

**Response to Reviewer #2:**
This study describes the first structures of FMDV-antibody interactions using mAbs isolated from a natural bovine host and shows the potential importance of the long HCDR3 in bovine antibodies against FMDV. One of the mAbs (isolated after sequential infection with two different serotypes of the virus) is able to neutralize both of these serotypes. This is a breakthrough in the field of FMDV (cross-reactive mAbs are usually non-neutralizing) and may be due to the long HCDR3 allowing binding into a depression which may not be so accessible to the murine mAbs historically used to map the antigenicity of FMDV. This is novel and important information for both FMDV and for bovine antibodies which is comprehensively described and generally well supported by the data.
**Response:** We appreciate the encouraging comment to our work.

Generally, well written but parts of this paper are difficult to follow. Several parts of the story require reference to the 20 items of supplementary material, why can’t more of these be included in the main body?

**Response:** Thanks for your suggestion. we have moved some figure of supplementary material to the main body, for example, S3 Fig and S11 Fig.

**Part II – Major Issues: Key Experiments Required for Acceptance**

R50 induced virus dissociation: What are the control conditions, I think the controls for this should include other neutralizing mAb/scFv which presumably will not induce dissociation? See additional comments on this in the minor issues section.

**Response:** FMDV neutralization by B77 is facilitated by blocking virus-receptor interaction via steric hindrance. And When we incubated FMDV-OTi with B77 at a molar ratio of 1:240 for 30 min or 60 min at 4 °C, the negative stain electron micrographs showed that B77 did not induce significant decrease of particle density after incubation for one hour. a micrograph has been added to S9 Fig.

**Part III – Minor Issues: Editorial and Data Presentation Modifications**
Reviewer #2: Introduction

It is suggested that bovine antibodies with ultralong HCDR3 may be better for producing high-affinity and broadly neutralizing antibodies. I understand this was the case for bovine antibodies identified to have broadly neutralizing activity against HIV but what is the general basis for the advantage?

**Response:** In term of HIV, the CD4bs is recessed and occluded on the native trimer, which greatly hinders access by human neutralizing antibodies and thereby renders the CD4bs effectively immunoquiescent in humans. The long HCDR3 of cow antibodies are nonetheless able to easily access the CD4bs on the trimer, therefore rendering this region immunogenic in the context of the cow antibody repertoire.

The general basis for the advantage：Human neutralizing antibodies usually have a short HCDR3 and generally flat binding surfaces. Antigenic sites recognized by human monoclonal antibodies are usually surface-exposed. Bovine antibodies with ultralong HCDR3 structures have the advantage to bind to epitopes that are not easily accessible to common antibodies with flatter paratope features.

Can an antibody with longer HCDR3 make contact with a conserved epitope comprising more distant amino acids, or perhaps tolerate more variation in the positioning of components of a conserved epitope?

**Response:** To our knowledge there have been no studies thus far whether longer HCDR3 can make contact with a conserved epitope comprising more distant amino acids or perhaps tolerate more variation in the positioning of components of a conserved epitope. We presume that longer HCDR3 have the advantage to tolerate more variation in the positioning of components of a conserved epitope.

It is proposed that this study will 'inform a structure-based rationale to design a universal FMDV vaccine'. I wondered if the authors would like to elaborate on this rationale, could this be mentioned in the discussion?
**Response:** Thanks for your suggestion, a description has been added in discussion section. **“**The novel neutralizing antigenic site formed by _OTi-VP1_V50, _OTi-VP1_D52, _OTi-VP1_P94, _OTi-VP1_E95, _OTi-VP1_L159(or _AWH-VP1_L157), _OTi-VP1_P160 (or _AWH-VP1_P158) and _OTi-VP3_D173 (or _AWH-VP3_D174) is highly conserved among FMDV serotypes O and A (Figs 6B and 6C). Meanwhile, Antibody (R50) targeting the novel antigenic site can induce a broad-spectrum neutralizing response against diverse isolates of serotype O and serotype A. Therefore, this could be a target for development of universal vaccine**”**

Isolation of antibodies from bovine plasmablasts
Fig 1A I don’t understand the gating strategy based on the text in the results section and the figure legend. Please can this be explained more clearly? For example, the schematic in the figure splits into ‘FMO’ and ‘sample’ what are these?

**Response:** The gating strategy aims to sort single O_FMDV^+^ A_FMDV^+^B cell that experiences isotype-switch and becomes expected IgG isotype. Unfortunately, the commercial anti-bovine IgG fluorescence antibody that can be used for flow cytometry was not available. To circumvent this roadblock, we designed the current gating strategy. First, the primary IgM^+^B cells were excluded, and then the O_FMDV^+^ A_FMDV^+^B cell from the IgM^-^B cells population were selected for sorting.

The O_FMDV^+^ A_FMDV^+^B cell are extremely rare population (approximately 25 per million PBMCs in our data) and more than 4 million PBMCs need to be loaded to sort the rare cells. Excellent fluorescence compensation setting and guarantee of specific binding are necessary for successful sorting. So we introduce the ‘FMO’ tube staining, in which except bait antigens are absent, all fluorescence antibodies are added. For ‘sample’ tube staining, all the reagents including the two bait antigens were used. By comparing the gates P4, P5 and P6 between the plots of ‘FMO’ tube and ‘FMO’ tube, we can determine the correct positions of P5 and good bind-specific of these selected staining regents. For clarity, we add detail explain for ‘FMO’ and ‘sample’, see the legends of Figure 1. For staining the B cells, we added two bait antigens (fluorescence labeled O_FMDV particle and A_FMDV particle), and the double positive O_FMDV^+^ A_FMDV^+^B cells (binding both O_FMDV and A_FMDV) derived antibodies should be broad reactivity to both FDMV serotype A and O. Subsequently, broad neutralizing antibody R50 was picked from these antibodies.

It would also be interesting to provide brief summary in the main text of how the animals were infected, how many B cells were sorted, what proportion were FMDV-specific and how many FMDV reactive B cells were characterized in order to find the three mAbs described.

**Response:** Concerning information of infection bovine and sorting B cells, we added brief description. Please see them in the main text.

Can this information be extrapolated to discuss the proportion of cross-reactive antibodies that could be expected in the polyclonal response?

**Response:** As revealed in data of FACS, the cross-reactivity B cells are extremely rare, displaying 21 per million PBMCs. To be note, the polyclonal response in this study was the results of multiple vaccination of bovine. To address this problem, further study on surveying the single vaccination with one FMDV serotype and identifying the proportion of double positive B cells in the PBMCs are needed in the future research.

How do the neutralizing titers of the three scFv compare with the range of titers generally expected for FMDV reactive mAbs?

**Response:** We used 100 TCID_50_ virus/well in the virus neutralization test to check the neutralization activity of B77, F145 and R50 scFv, An neutralizing titers (NT) value of 100 µg/ml was used as a cutoff for neutralization, and >100 µg/ml was defined as showing no virus-neutralizing activity. For the FMDV reactive mAbs, we found obvious cytopathic effect, even the concentration of mAb reached to 500 µg/ml. The NT value of B77, F145 and R50 scFv are all less than 50 µg/ml. Accordingly, we determine these tree scFv are neutralizing antibodies.

Overall architectures of the FMDV-NAb complexes
Three copies of B77 and F145 clearly bind to sites located closely around the 3-fold axis. R50 is described as binding near the quasi-5-fold axis, but it doesn’t seem to me to be particularly near to the 5-fold or really any symmetry axis. If anything, the closest feature seems to be the pentamer-pentamer boundary across the 2-fold and this is also where the signal is seen in the maps. So I don’t really understand why the R50 binding site is described as near the 5-fold and chosen to be displayed as binding round the 5-fold. Perhaps this should be explained?
**Response:** Thanks for your suggestion, a description has been modified in Overall architectures of the FMDV-NAb complexes section. " By contrast, R50 scFv binds to a depression between the 2- and 5-fold axes of FMDV-OTi and FMDV-AWH. "

Interaction of B77 and F145 with FMDV-OTi
Neutralization is suggested as being by steric hindrance to integrin receptor binding. Can the authors comment on the receptor usage of these viruses, they are grown in BHK cells suggesting they are cell culture adapted to use heparan sulphate so may not require integrins, would this change the interpretation?
**Response:** In the cellular entry of FMDV, HS (co-receptor) only fulfill this role in the initial contact of virus to cells, but viral internalization must be dependent in integrin receptor. B77 and F145 block the virus-receptor interaction via steric hindrance, furthermore hinder viral internalization.

Determinant for the serotype O-specificity of F145 and B77
Lines 209-219: Apologies but I could not understand all of this section. The rationale for this section is not clear. Are you creating new ‘artificial escape mutant viruses’ here by reverse genetics? The text says into FMDV-AWH but then in the rest of the section only the O virus is described… I think this section needs to be made more clear.
**Response:** We have modified a description in Determinant for the serotype O-specificity of F145 and B77 section. **“**For B77, we identified that the neutralization escape mutation _OTi-VP2_N190→_AWH-VP2_S190 enables the escape of antibody-mediated neutralization (virus neutralization [VN] titer >400 µg/ml) (Fig 3C and S9 Table). For F145, we mutated FMDV-OTi amino acid by FMDV-AWH’s sequence at the corresponding positions (S72D, N190S and T191S), rescued virions by reverse genetics approach and evaluated the neutralization capacity using virus neutralization test (VNT).

FMDV-OTi-R50 and FMDV-AWH-R50 interfaces
Figures 4 and 5 are beautiful but if the orientation of the structure must be changed between the parts of the figure it would help if this was pointed out (e.g. the view of the enlarged inset panels is rotated).

**Response:** Thanks for your suggestion, an arrow description has been added to Figs 4A and 5A.

R50 induced virus dissociation: how does the molar ratio of R50 used for dissociation compare with the concentrations of R50 used in the neutralization experiments?

**Response:** We used 100 TCID_50_ virus/well in the virus neutralization test to check the neutralization activity of R50 scFv, the NT value of R50 scFv are all less than 50 µg/ml. The virus capsid of FMDV comprises 60 copies each of four virus-encoded proteins, VP1, VP2, VP3 and VP4. Structures of virus-NAb complex reveal R50 engages the capsids of both FMDV-O and FMDV-A between the 2- and 5-fold axes and each virus particle binds to 60 R50 scFv antibodies. we incubated FMDV-OTi with R50 at a molar ratio of 1:240 for 30 s or 60 s at 4 °C, the negative stain electron micrographs showed that R50 destabilized the virus particle and induced dissociation of virus particles into capsid pentamers, and very few particles remained intact after 60 s. Meanwhile we also incubated FMDV-OTi with R50 at a molar ratio of 1:60 or 1:120, very few particles remained intact after 60s.

What are the control conditions, I think the controls for this should include other neutralizing mAb/scFv which presumably will not induce dissociation?

**Response:** FMDV neutralization by B77 is facilitated by blocking virus-receptor interaction via steric hindrance. And When we incubated FMDV-OTi with B77 at a molar ratio of 1:240 for 30 min or 60 min at 4 °C, the negative stain electron micrographs showed that B77 did not induce significant decrease of particle density after incubation for one hour. a micrograph has been added to S9 Fig.

### The EM images are not high enough magnification to see the presence of the highlighted pentamers, could an enlarged inset be used to convey this point? Not a requirement but sucrose density gradient analysis would provide more positive evidence for dissociation into pentamers.

### Response: We raised the magnification to 150KX, and found the pentamers and an enlarged inset have been added to S9 Fig.

### A few words to introduce the dissociation of FMDV would also be useful and perhaps discussion somewhere of other mAbs with similar properties against FMDV or comparable destabilization of other picornaviruses would also be useful. Response: Thanks for your suggestion，a description has been added to FMDV-OTi-R50 and FMDV-AWH-R50 interfaces Section. “Previous studies have reported that A9-Fab destabilizes EV71 virions and as much as 70% of EV71 F-particles had been destroyed when incubated with A9-Fab at 37°C.”

### Fig S5 seems to have a problem with poor quality plaque assay images.

**Response:** we apologize for the poor quality image presented, We reconducted the plaque assay. And the results were substituted into Fig S5.

**Response to Reviewer #3:**

He et al. characterized foot-and-mouth disease virus (FMDV) and three neutralizing antibodies (NAbs). The authors isolated two NAbs for FMDV strain O and isolated one NAb that is cross-reactive for strains O and A. By means of cryogenic electron microscopy (cryo-EM), He et al. determined high-resolution structures of four FMDV-NAb complexes. They also performed neutralization experiments and generated neutralization escape mutants. Presumably to make experiments simpler, they made and used single-chain fragment variable antibodies. The two O-strain NAbs (B77 and F145) bound FMDV near the 3-fold symmetry axis of the capsid. The O/A cross-binding NAb (R50) bound FMDV near the 5-fold symmetry axis. R50 bound most of the same residues in both strains. Precise interactions of the NAbs and FMDV were mapped. Escape mutants were successfully produced. The authors proposed neutralizing mechanisms for all three NAbs. The analysis is thorough and rigorous. Structural experiments were complemented by mutational experiments. Antibody-producing cells were obtained from an animal by an elaborate protocol. Conclusions are backed up by substantial evidence. I found the work enlightening and a step forward for the field. This work has implications not only for FMDV work, but also for virus-antibody studies in general. It shows an example of how a cross-serotype neutralizing antibody binds to a virus. At least two different neutralizing mechanisms appear to be at work because the R50 caused different effects on FMDV than did the other two NAbs. I recommend publication but suggest that the following corrections be made.

**Response:** We appreciate the encouraging comment to our work.

He al. proposed mechanisms for how the three NAbs neutralize FMDV. B77 and F145 are proposed to block receptor binding, and R50 is proposed to cause the FMDV capsid to fall apart. The latter was discovered through an interesting observation by the authors. Treatment of FMDV with R50 resulted in dissociation of the particles. I have several questions:

-For picornavirus capsids, a "promoter" was defined as one closely associated VP1-4 subunits and is considered an assembly unit (5 protomers form a pentamer, then 12 pentamers form a capsid). I note that the R50 interaction is an inter-protomer interaction (Figs. S8 and S10). The authors should mention this. Does that fact have any implications for the mechanism of neutralization?

### Response: We have added a description to abstract and discussion Section. “In contrast, R50 engages the capsids of both FMDV-O and FMDV-A between the 2- and 5-fold axes and binds to the BC/EF/GH-loop of VP1 and to the GH-loop of VP3 from two adjacent protomers, revealing a previously unknown antigenic site.” “The structures of FMDV-OTi and FMDV-AWH with a cross-serotype NAb R50 exhibit a novel neutralizing antigenic site located in a shallow depression on the capsid, contacting two major capsid proteins VP1 and VP3 from two adjacent protomers.”

Implications: R50 binds to the BC/EF/GH-loop of VP1 and to the GH-loop of VP3 from two adjacent protomers. And two adjacent protomers are intra-pentamer other than inter-pentamer. FMDV-OTi or FMDV-AWH incubated with R50 at a molar ratio of 1:240 for 30 s or 60 s at 4 °C, R50 destabilized the virus particle and induced dissociation of virus particles into capsid pentamers. So, the neutralization mechanism of R50 is promoting the dissociation of FMDV virions before infection.

-Yes, the binding of B77 or F145 will occlude the receptor binding site. But in vivo, one is not likely to have all 60 sites occupied. Therefore, presumably the receptor could simply bind to an unoccupied site. Also, in vivo the antibody will be an IgG or some other multi-site entity. What alternative mechanism does these facts suggest?

### Response: For B77 or F145, In vivo the antibody will be an IgG or some other multi-site entity, so mechanisms of viral neutralization may also be aggregation of virus particles except for blocking virus-receptor interaction via steric hindrance. a description has been added to “Interaction of B77 and F145 with FMDV-OTi” Section. “Meanwhile, in vivo the antibodies are IgG molecules or some other multi-site entity, a situation that could result in aggregation of virus particles if one virus particle becomes crosslinked with another. Aggregation of virus particles can cause a reduction in the amount of infectious virions and may result in the neutralization.”

Could antibody binding prevent conformational changes during disassembly/uncoating as was hypothesized for a neutralizing antibody to poliovirus?

**Response:** Several studies have previously shown presumed uncoating intermediate of poliovirus, and antibody binding (PVSP6A and PVSP29F) can prevent conformational changes during disassembly/uncoating. But to our knowledge there have been no studies thus far showing an uncoating intermediate particle from the FMDV infection pathway. So, we don't know for sure if antibody binding prevents conformational changes during disassembly/uncoating.

-Are the B77 and F145 interactions inter- or intra-protomer?

### Response: We have added a description to discussion Section. “The structures of FMDV-OTi in complex with serotype O-specific NAbs F145 and B77 show that the binding sites (BC-loop and HI-loop of VP2) are also surface-exposed and intra-protomer sites.”

-The dissociation might explain the lower particle density seen in the micrographs of frozen FMDV-R50 complexes (Fig. S1). Cryo-samples of FMDV-R50 were prepared after 30 sec incubation. Is the lower density in the micrographs further evidence of the dissociation?

### Response: we appreciate your insightful comment, a description has been added to FMDV-OTi-R50 and FMDV-AWH-R50 interfaces Section. “Meanwhile, the cryo-electron microscopy (cryo-EM) micrographs also showed significantly lower particle density when incubated for 30s (S1 Fig).”

-In those cryogenic-samples were pentamer-R50 or pentamer particles seen? Does R50 retain affinity after the dissociation?

**Response:** We find pentamer-R50 or pentamer particles in negative stain electron micrographs. a micrograph has been added to S9 Fig. R50 binds to the VP1 and VP3 from two adjacent protomers within a pentamer. The R50 binding sites are not destroyed after the dissociation. So, we presumed R50 retain affinity after the dissociation.

The authors note five known FMDV neutralizing antigen sites on FMDV (pp. 12-13). The R50 binding site appears to be a new site. This should be stated. Do B77 and F145 bind to one of the five sites or to a different site? The five sites are all intra-protomer sites. The R50 site is novel in that it overlaps the protomer boundary.

### Response: we appreciate your suggestion; a description has been added to discussion Section. “For F145, neutralization escape mutation and reverse genetics reveal that VP2 S72 within antigenic site 2 plays the most essential roles in the serotype O-specific neutralization activity. For B77, the key epitope residue on VP2 N190 is the major determinant for serotype O-specific recognition.VP2-190 around the threefold axis is a new neutralizing epitope, which is close to antigenic site 2. Bovine antibodies with ultralong HCDR3 structures have the advantage to bind to epitopes that are not easily accessible to common antibodies with flatter paratope features. The structures of FMDV-OTi and FMDV-AWH with a cross-serotype NAb R50 exhibit a novel neutralizing antigenic site located in a shallow depression on the capsid, contacting two major capsid proteins VP1 and VP3 from two adjacent protomers.”

The A serotypes have a proline at residue 47 of VP1 (Fig. 5E). The O serotypes have S or Q at that position. Do the prolines induce a different loop conformation for the A serotypes or is the conformation similar despite the presence of a proline in the A serotypes? Apparently the conserved residues at 50 and 52 still bind to the antibody.

**Response:**

**Superposition of FMDV-OTi and FMDV-AWH.** VP1 BC loop of FMDV-OTi (gray) and FMDV-AWH (blue) are aligned and shown as cartoons in the same orientation. _AWH_P47 and _OTi_Q47 are displayed as sticks with the same color scheme as in the main chain.

The structural alignment of FMDV-OTi and FMDV-AWH capsid proteins show that the proline does not induce substantial conformational changes in the A serotype VP1 BC-loop.

A figure in the main part of the paper that shows epitopes on unbound FMDV would be very helpful. I recommend showing FMDV capsid proteins as ribbons.

**Response:** we appreciate your suggestion; we have supplemented a Figure 6 in the main part of the paper.

I suggest the NAb footprints, now shown in Fig. S3, be put into Fig. 2.

**Response:** we appreciate your suggestion; we have put Fig.S3 into Fig2

Many details of the FMDV-NAb interactions are written out in the text (pp. 7-11). Much of this would be clearer if it were put in a table and the pertinent messages about the interactions described in the text.

**Response:** Thanks for your suggestion, the details of the FMDV-Nab interactions have been supplemented in Table S4-S7 and the pertinent messages about the interactions also have been described in the text.

In Tables S4-S7, interacting residues are given for all four complexes. In each case, red designates "a hydrogen bond or salt-bridge". What are the black interactions? Many or most of the black ones are polar residues (N or O) and the distance is less than 4 angs. Seemingly these would also qualify for being H-bonds or "salt" interactions?

**Response:** We focus on the analysis of the hydrogen bond/salt-bridge between the amino acid side chains (red). The black ones (N or O) are in the main chain, we presume they would not significantly alter the interaction pattern. A detail description has been added to Tables S4-S7.

I was confused by the description of the grids that were used.

-"carbon-coated copper grid" was stated but in parentheses was stated "GIG Au 1.2/1/3". Was gold or copper used?

-I suggest instead of "Lantuo" that the full company name be stated for the benefit of those who are unfamiliar with that company. (This was the first time I had heard of it.)

### Response: we apologize for the confusing description; we have supplemented the detailed information in Methods section. “A 3 μl aliquot of the mixture was applied to a glow-discharged carbon-coated gold grid (GIG, Au 1.2/1.3, 200 mesh; Jiangsu Lantuo Biotechnology Co. Ltd., China).”

He et al. states they used a calibrated magnification of 110kX. How was magnification calibrated? Was magnification refined with the crystal structure of FMDV that was fitted into the cryo-EM density maps?

**Response:** A small pixel size helps to reduce the information loss, which provides high-resolution images. Arctica pixel size is shown as follow.

**Arctica Pixel Size**

| **Magnification** | **Pixel size_a** | **Pixel size_b** | **Pixel size** |
| --- | --- | --- | --- |
| BM-Falcon_110000X | 93.2 pm | 93.2 pm | 0.932 Å |
| BM-Falcon_78000X | 127 pm | 127 pm | 1.27 Å |
| BM-Falcon_53000X | 192 pm | 192 pm | 1.92 Å |
| BM-Falcon_39000X | 269 pm | 269 pm | 2.69 Å |
| BM-Falcon_23500X | 409 pm | 409 pm | 4.09 Å |

In order to obtain high-resolution image,we select a pixel size of 0.932 Å corresponding to a calibrated magnification of ×110 kX. The crystal structure of native FMDV O1BFS (PDB:1BBT) was manually placed into the cryo-EM map for FMDV particles and rigid-body fitted with UCSF Chimera.

Some figures are too small to see data clearly (e.g., Figs 3, S1, S2, S9).

**Response:** Thanks for your suggestion, a modification has been added to Figs 3, S1, S2, S9.

The following terms or portions of the paper are unclear or need to be fixed:

-"quasi 5-fold axis" (p. 6), I know of no quasi 5-fold axis in picornaviruses.

### Response: We apologize for our inaccurate writing. a description has been modified in abstract section. “R50 engages the capsids of both FMDV-O and FMDV-A between the 2- and 5-fold axes”

-"the densities of the bound NAbs away from the contact surface were not sufficiently clear to trace all main chains (S2 Fig)." I do not see this illustrated or discussed in Fig S2.

**Response:** We have added a description to S2 Fig section. **“**Black dashed circles show the densities of the bound NAbs away from the contact surface. The densities are not sufficiently clear to trace all main chains.**”**

-"The globular knob of F145 interacts with VP2, but the globular knob of R50 binds to a shallow depression on the capsid, contacting the two major capsid proteins VP1 and VP3 (Figs 2I-2L)." I do not see this in those panels. Perhaps a label or different figure is needed?

**Response:** Thanks for your suggestion. a figure has been added to S3 Fig.

-Why is Fig. S4 referenced at the top of p. 8? It follows the comment ". . . is strictly conserved among serotype O".

**Response:** S4 Table and Fig 3C are referenced to **“**. . . is strictly conserved among serotype O**”.** And S4 Fig is referenced to **“**The structural alignment of FMDV-OTi and FMDV-AWH capsid proteins showed no substantial conformational changes in the VP2 BC-loop and HI-loop, which are the epitopes of B77 and F145**”.**

-p. 8, line 198, "F45"

**Response:**. F45 has been modified as F145.

-p. 10, lines 252-254. The sentence here refers to Figs 5A-5D, but I don't see hydrogen bonds shown in those figures.

**Response:** Figs 5A-5D has been modified as Fig 5A and S7 Table.

-p. 16, line 410, I think Supplemental Table 1 is what the authors intended.

**Response:** Thank you for pointing out this mistake. Supplemental Figure 1 has been modified as Supplemental Table 1.

-In Fig. S1, FSC Corrected, FSC Unmasked, and FSC Masked need to be explained.

**Response:** We have added a description to S1 Fig. **“**FSC curves are plotted before (gray) and after (yellow) masking in addition to post correction (orange), accounting for the effect of the mask using phase randomization.**”**

-In Fig. S1, "Typical electron micrographs were collected with a defocus of ...." Specific defocus values for specific cryo-samples are given. This suggests all micrographs for each specified sample were collected only at those values. The methods say a range of defocus values were used.

**Response:** Each micrograph has a specific defocus value, but different micrograph may have different defocus and defocus values are all at a range of −2.4 to −1.4 μm (For F145，we collected 1216 micrographs).

-Fig. S3 "Roadmap" should be placed in parentheses because it is a jargon term. Was the Roadmap program used? No reference or explanation was given for how these diagrams were determined.

**Response:** We have modified a description in Figs 2I-2L. **“**Footprints of B77, F145 and R50 on the FMDV surface. The figure shows a 2D projection of the FMDV surface produced using RIVEM[1]. Five-, 3-, and 2-fold icosahedral symmetry axes are marked as pentagons, triangles, and ovals, respectively, on one icosahedral asymmetrical unit. The spherical polar angles (θ, ϕ) define the location on the icosahedral surface. The depictions are radially depth cued from blue (radius = 130 Å) to red (radius = 155 Å). The residues in the scFv footprints are shown in purple.**”**

-In many places, authors call ribbon depictions of proteins "cartoons" or "cartoon representations". "Ribbon diagram" is a more accurate term.

**Response:** Thanks for your suggestion. cartoon has been modified as Ribbon diagram.

-In caption for Fig. 1, "(B) . . . are green." Nothing in Fig. 1B is green.

**Response:** We are very sorry for our incorrect writing. a description has been modified in Fig.1. **“**NT values of 0-10 µg/ml are red, 10-25 µg/ml are orange, 25-50 µg/ml are blue, and ˃100 µg/ml are white.**”**

-Figs. 4 and 5, "labels in the magenta regions" and "labels in the pink regions" is unclear wording.

**Response:** We have modified a description in Fig.4 and Fig.5. **“**Surface representation of FMDV-OTi-R50 showing the interaction interface between R50 (ribbon diagram) and the capsid. The interface is framed and shown as a close-up view in the right panel. The residues in R50 responsible for interacting with the capsid are shown as yellow sticks. The interface amino acid residues in VP3 **(B)** and VP1 **(C)** are colored magenta and labeled; the interface residues in R50 are colored yellow and labeled **(D)**.**”**

-Fig. 5, "... indicated by blue and white ..." Do the authors mean light blue?

**Response:** We are apologize for inaccurate writing. Blue and white has been modified as light blue.
